# Cardiac Autonomic and Cardiac Vagal Control During and After Depressive and Happiness Autobiographical Memories in Patients With Major Depressive Disorder

**DOI:** 10.3389/fpsyt.2022.878285

**Published:** 2022-06-02

**Authors:** I-Mei Lin, Yin-Chen Wu, Wen-So Su, Chiao-Li Khale Ke, Pei-Yun Lin, Mei-Feng Huang, Yi-Chun Yeh, Kuan-Ta Wu, Cheng-Fang Yen, Chih-Hung Ko, Sheng-Yu Fan

**Affiliations:** ^1^Department of Psychology, College of Humanities and Social Sciences, Kaohsiung Medical University, Kaohsiung, Taiwan; ^2^Department of Medical Research, Kaohsiung Medical University Hospital, Kaohsiung, Taiwan; ^3^Department of Psychiatry, Kaohsiung Medical University Hospital, Kaohsiung, Taiwan; ^4^Department of Psychiatry, Kaohsiung Municipal SiaoGang Hospital, Kaohsiung Medical University, Kaohsiung, Taiwan; ^5^Department of Psychiatry, School of Medicine, College of Medicine, Graduate Institute of Medicine, Kaohsiung Medical University, Kaohsiung, Taiwan; ^6^Department of Psychiatry, School of Medicine, College of Medicine, Kaohsiung Medical University, Kaohsiung, Taiwan; ^7^Graduate Institute of Medicine, College of Medicine, Kaohsiung Medical University, Kaohsiung, Taiwan; ^8^Health Management Center, Kaohsiung Medical University Hospital, Kaohsiung Medical University, Kaohsiung, Taiwan; ^9^Institute of Gerontology, College of Medicine, National Cheng Kung University, Tainan, Taiwan

**Keywords:** cardiac autonomic, cardiac vagal control, depression, heart rate variability, respiratory sinus arrhythmia

## Abstract

**Background:**

Heart rate variability (HRV) and respiratory sinus arrhythmia (RSA) are indices of cardiac autonomic and cardiac vagal control (CVC), both of which are markers of emotional regulation and physical health. This study examined (1) the differences in cardiac autonomic regulation and CVC during baseline, depressive, and happiness autobiographical memory tasks between participants with major depressive disorder (MDD group) and healthy controls (HC group); (2) the associations between depressive symptoms and cardiac autonomic and CVC; and (3) the reactivity and recovery of cardiac autonomic and CVC between the MDD and HC groups.

**Methods:**

A total of 168 and 178 participants were included in the MDD and HC groups, respectively. Demographic data and the Beck Depression Inventory-II were collected before the experimental procedure. Lead II electrocardiograph (ECG) was measured during baseline, depressive, and happiness autobiographical memory tasks, and then interbeat intervals from ECG were converted to the time and frequency domains of HRV and RSA.

**Results:**

The participants in the MDD group showed lower HRV (including standard deviation of normal to normal intervals, low frequency, the natural logarithm of low frequency, and the natural logarithm of high frequency) and CVC (RSA and lnRSA) than those in the HC group. Depressive symptoms were positively correlated with heart rate and negatively correlated with the indices of cardiac autonomic and CVC. There was significantly increased reactivity and recovery of cardiac autonomic and CVC during and after depressive and happiness autobiographical memory tasks in the HC group, but not in the MDD group.

**Discussion:**

Participants with MDD had cardiac autonomic and CVC dysregulation, decreased reactivity, and did not recover to baseline after emotional provocations. These results can be the theoretical basis for clinical intervention by using HRV biofeedback to restore cardiac autonomic regulation and CVC during and after emotional events in the future.

## Introduction

Dysregulation in cardiac autonomic and cardiac vagal control (CVC) includes sympathetic nervous system (SNS) hyperactivity and/or parasympathetic nervous system (PNS) hypoactivity, also called vagal withdrawal (1). This dysregulation is widely viewed as an index of capacity, adaptation, and flexibility in emotional regulation in patients with major depressive disorder [MDD; (1–3)]. Heart rate variability (HRV) and respiratory sinus arrhythmia (RSA) were measured using electrocardiography (ECG), which has the advantages of being noninvasive, cost-effective, continually tracking the changes in different situations, and is a common biomarker of cardiac autonomic regulation and CVC (4–6).

Two pathophysiological mechanisms of cardiac autonomic regulation and CVC are present in MDD. First, SNS hyperactivity (4–6), one study reported lower interbeat intervals (IBIs), frequency/high frequency (LF/HF) ratios, and higher heart rates in 498 unmedicated patients with MDD than that in 462 healthy controls (HC group; (7)). Second, for PNS or vagal withdrawal, two meta-analysis studies reported lower resting HRV in the MDD group than in the HC group (8, 9). Kemp et al. included 18 case-controlled studies and found a reduced resting high frequency (HF) of HRV in 401 patients with MDD compared to 407 HC (8). Koenig et al. included six studies that included 99 MDD and 160 HC, and the results found reduced resting HF of HRV in the MDD group (9). Moreover, some original studies also reported lower low frequency (LF) HRV (7), HF (7, 8, 10–12), a standard deviation of normal to normal intervals (SDNN), root mean square of the successive differences, number of pairs of successive NNs that differ by more than 50 ms (NN50), and the percentage of NN50 (11–14) in the MDD group than in the HC group.

Two theories link cardiac autonomic, CVC, and emotional regulation. First, polyvagal theory (15) states that appropriate physiological and behavioral states can facilitate prosocial behavior. Second, the neurovisceral integration model (16) indicates that the vagus nerve integrates the prefrontal cortex and heart rhythm and that people have positive outcomes in executive functions, emotion, and health. However, these theories focus on the resting baseline and do not explore cardiac autonomic regulation and CVC under different situations. Recently, the vagal tank theory (17) emphasized CVC under different situations, including resting, reactivity, and recovery, and extended this theory to neurophysiological, cognitive, and social interactions. The reactivity of cardiac autonomic and CVC refers to the changes in HRV or CVC values from a resting baseline to an experimental/emotional task. The recovery of cardiac autonomic and CVC represents the change from an experimental/emotional task to a period after that event. The vagal tank theory hypothesizes that in the process of adaptation and emotional regulation, cardiac autonomic and CVC values may decrease or increase during the event and then cause faster or longer cardiac autonomic and CVC values to return to the resting baseline (4, 6, 18, 19).

Regarding resting HRV and RSA values, patients with MDD had reduced vagal modulation at the resting baseline (20, 21). Rottenberg et al. indicated that low CVC was related to decreased cardiac outflow, low HRV, decreased social engagement, and a lack of flexibility to cope with environmental demands (22). Patients with MDD tend to exhibit inactivity, flat facial expression, lack of gaze behavior, less eye contact in social interaction, and constricted and stereotyped emotional responses in laboratory situations (22, 23). Rottenberg's (6) meta-analysis found a reduced resting CVC in 262 patients with MDD compared to 334 HC. Licht et al. (24) found a lower RSA and SDNN in the 1,849 Netherlands MDD group than in the 524 HC group. Some studies have found that low RSA and HF of HRV are related to emotional dysregulation and social dysfunction in MDD when they counter social interaction (6, 19, 24). Therefore, the aforementioned studies support higher SNS, lower PNS, and lower CVC during the resting baseline in the MDD group than in the HC group (1, 13).

Some studies have used a cognition task (e.g., challenging task, speech task, or mirror tracing task) or a sad video to examine the reactivity and recovery of HRV and RSA related to cardiac autonomic regulation and CVC. Cyranowski et al. (25) used a speech task to decrease CVC activation and a relationship imagery task to increase CVC activation. The results showed CVC withdrawal during the speech task and a lack of recovery to baseline after 1 h of the speech task in the MDD group. Furthermore, the MDD group did not show increased CVC activation during the relationship imagery task. These results indicated reduced cardiac autonomic and CVC during a cognition task in both MDD and HC groups. The participants in the HC group recovered to the baseline, but patients with MDD did not recover to the baseline, which was related to their vagal withdrawal.

Other studies have reported atypically increased PNS or CVC and decreased SNS during or after an experimental task in the MDD group. Shinba (10) compared 22 drug-naïve depressed patients with 47 age- and sex-matched HC groups at baseline, during, and after a cognitive challenge task. The results showed lower HF and higher LF/HF ratio during the challenging task in the HC group but not in the MDD group. This result indicated decreased PNS regulation and increased SNS activation during cognitive stress in the HC group but not in the MDD group. Rottenberg et al. (26) showed a sad video to the MDD and HC groups; participants who were crying after watching a sad video had increased RSA (CVC) in the HC group, which was called vagal rebound. Rottenberg et al. (18) also found vagal rebound after speech and mirror tracing tasks in the HC group. However, the vagal rebound has not been observed in patients with MDD. These phenomena are considered flexible or adaptive (19), and evidence indicates that moderate reactivity of autonomic and CVC is most adaptive, while too less or too much reactivity may be considered maladaptive (27).

Patients with MDD frequently ruminate or recall negative events and experience depressive moods. Previous studies have indicated the positive autobiographical memory deficit that patients with MDD have difficulty in recalling the positive events in their daily life (28). Given that the examined reactivity and recovery of the cardiac autonomic regulation and CVC during and after social interactions is relatively new, especially for participants' depressive and happiness autobiographical memories.

The aims of this study were as follows: (1) to investigate the group differences in cardiac autonomic regulation and CVC during baseline between the MDD and HC groups; (2) to examine group differences in the reactivity and recovery of cardiac autonomic and CVC during depressive and happiness autobiographical memory tasks in the MDD and HC groups; and (3) to examine the association between depressive symptoms and indices of cardiac autonomic and CVC. We hypothesized that participants' flexibility or fluctuation of cardiac autonomic and CVC were impaired in the MDD group but not in the control group. Therefore, decreased reactivity of HRV and RSA indices, and faster recovery after depressive and happiness autobiographical memory tasks were found in the HC group, but not in the MDD group. There were negative correlations between depressive symptoms and cardiac autonomic and CVC indices.

## Materials and Methods

### Participants

Participants were recruited from the outpatient clinics of the Department of Psychiatry at Kaohsiung Medical University Hospital, Kaohsiung Municipal SiaoGang Hospital, Kaohsiung Municipal Ta-Tung Hospital, and Kaohsiung Chang Gung Memorial Hospital. The inclusion criteria for patients with MDD were diagnosed by psychiatrists based on the *Diagnostic and Statistical Manual of Mental Disorders*, fifth version (29). Participants in the MDD group had no current or past physical illness (e.g., cardiovascular disease, cancer, liver, or kidney disease) and no severe psychiatric disorder except for MDD or MDD comorbid with anxiety disorder.

The participants in the HC group were recruited from the Health Management Center of Kaohsiung Medical University Hospital, college students at Kaohsiung Medical University, and the community of Kaohsiung city. The participants aged between 20 and 75 years, had no current or past physical illness (e.g., cardiovascular disease, cancer, liver, or kidney disease), and had no severe psychiatric disorder (e.g., schizophrenia, bipolar disorder, or dementia).

Institutional review board approval was obtained from the ethics committees of Kaohsiung Medical University Hospital and Kaohsiung Chang Gung Memorial Hospital, Taiwan, and informed consent was obtained from each participant before the study. Participants received approximately USD 11 (NT$300) as compensation for their participation.

#### Materials and Measurements

Demographic characteristics and the Beck Depression Inventory-II (BDI-II) scores were collected before the experimental procedure. Disease history and medications were obtained from medical records, and electrocardiography (ECG) was measured during the experimental stages for all participants.

The BDI-II is a self-report inventory that includes 21-item, four-point Likert questions to assess depressive symptoms in the period of 2 weeks before the study, such as sadness, pessimism, past failure, loss of pleasure, and feelings of guilt (30). The total score of depression ranged from 0 to 63, with two subscales, namely, somatic depression (e.g., fatigued) and cognitive depression (e.g., worthless) (30).

The ECG sensor with a sampling rate of 2,048 Hz was used to acquire lead II ECG raw signals (PQRST wave), and a respiration sensor with a sampling rate of 256 Hz was used to obtain the breathing rates. The ECG and breathing rates were collected using ProComp Infiniti™ version 6.1.1 (Thought Technology Ltd., Montreal, Quebec, CAN).

### Research Design and Experimental Procedure

**Figure 1:**
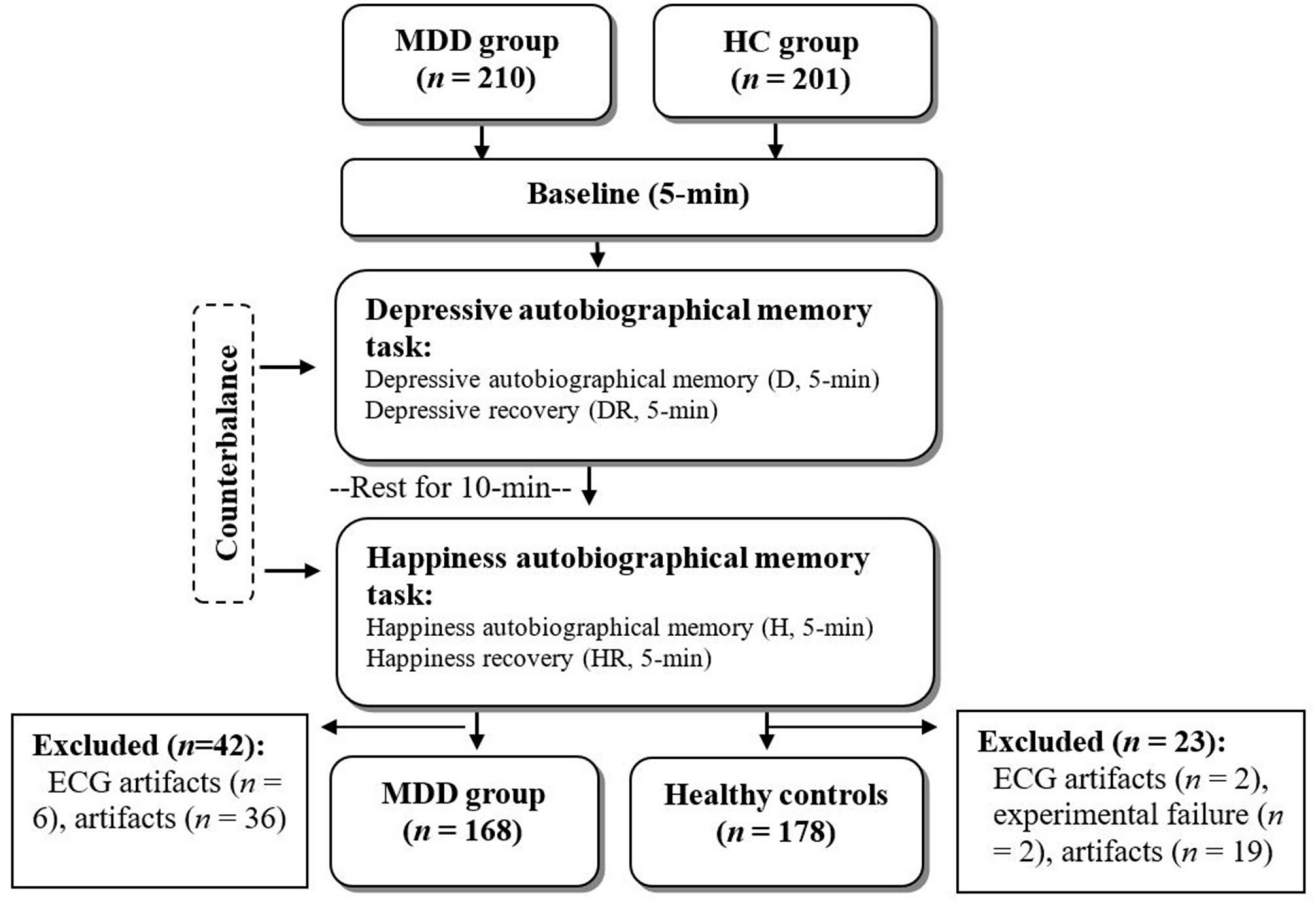
Study flow chat.

A cross-sectional study with a counterbalanced design was used to balance the sequence effect between depressive and happiness autobiographical memory tasks. The total experimental stage was 25 min, with eyes closed in the following sequence: (1) baseline (B, 5 min), in which participants sat on a comfortable chair and rested; (2) depressive autobiographical memory task (D; 5 min), in which participants were guided to recall their depressive autobiographical memory and maintain the depressive feeling in their mind, followed by a 5-min depressive recovery stage (DR), in which participants were asked to sit on a chair and rest; (3) happiness autobiographical memory task (H; 5 min), in which participants were guided to recall their happiness autobiographical memory and maintain the happy feeling in their mind, followed by a 5-min happiness recovery stage (HR), in which participants were asked to sit on a chair and rest (Figure 1); and (4) depressive and happiness autobiographical memory tasks, with a 10-min break in between.

Patients with MDD were instructed to take their medication as usual. All participants were asked to refrain from caffeine or alcoholic beverages and not exercise excessively or smoke 3 h before the experimental procedure. The psychophysiological measurements were arranged between 9 am and 5 pm in a sound-attenuated and temperature-controlled room.

### Data Reduction and Statistical Analysis

A CardioPro Infiniti HRV analysis module (Thought Technology Ltd., Montreal, Quebec, CAN) with a fast Fourier transform was used to analyze the raw ECG signals. The automatic filter to exclude IBI was more or <20% of the previous IBI. We used visual inspection to detect ECG artifacts and modified them by adding, splitting, and averaging consecutive IBI. If the total ECG artifacts were more than 20% in each experimental stage, these data were deleted from the statistical analyses. These IBI were converted to (1) the frequency-domain of the HRV: included LF power (0.04–0.15 Hz; influenced by both SNS and PNS activations and related to blood pressure regulation by baroreflexes), high frequency (HF) power (0.15–0.4 Hz; reflects vagal and PNS activities), and the LF/HF ratio; and (2) time-domain of the HRV: included the SDNN calculated from the IBI of the ECG signals and referred the total HRV (4, 31). The CVC index was acquired from the power spectrum at 0.13–0.5 Hz as RSA power (6, 18). The non-normal distribution of the frequency domain of HRV and RSA indices was transformed into natural logarithms as lnLF, LnHF, and lnRSA.

The HRV and RSA reactivities were calculated using the values during the depressive autobiographical memory minute baseline (D-B), as well as the values during the happiness autobiographical memory minute baseline (H-B). The HRV and RSA recoveries were calculated from the values during depressive recovery minute depressive autobiographical memory (DR-D), the values during happiness recovery minute happiness autobiographical memory (HR-H), the values during depressive recovery minute baseline (DR-B), and the values during happiness recovery minute baseline (HR-B).

Multivariate analysis of variance (*F-*test) and chi-square tests were used to examine the group differences in demographic characteristics, BDI–II, breathing rates, heart rate (HR), HRV, and CVC indices at baseline. Pearson's correlation was used to examine the associations between depressive symptoms, HRV, and CVC indices. One-way repeated measures ANOVA was used to examine the time-main effects on HRV and CVC indices during different experimental stages for the MDD and HC groups, respectively. Mauchly's test of sphericity with Greenhouse–Geisser adjustment was applied in one-way repeated measures ANOVA, and the Bonferroni method was used for *post-hoc* comparisons. All analyses were performed using SPSS predictive analytics software (version 21.0; SPSS Inc., Chicago, IL, USA).

## Results

### Participants' Characteristics and Research Variables

A total of 411 participants were enrolled, including 210 patients with MDD (MDD group) and 201 healthy controls (HC group). After deleting ECG artifacts in one of the experimental stages of ECG artifacts and experimental procedure failure, there were 168 participants in the MDD group (mean age, 42.72 ± 13.84 years; 122 women and 46 men) and 178 participants in the HC group (mean age, 40.26 ± 13.07 years; 112 women and 66 men) who had five stages of HRV and RSA data. There were no significant differences in age, sex, or body mass index between the two groups (Table 1).

**Table 1:** Demographic characteristics and research variables between the MDD and HC groups (*N* = 346).

**Variables**	**MDD group (*n* = 168)**	**HC group (*n* = 178)**	***F /χ^2^***	***p***
**Age**	42.72 (13.84)	40.26 (13.07)	2.89	0.090
**Sex**				
Female, *n* (%)	122 (72.62%)	112 (62.92%)	3.71	0.054
Male, *n* (%)	46 (27.38%)	66 (37.08%)		
**Body mass index**	23.68 (4.66)	23.56 (3.48)	0.07	0.787
**Medications**				
Antidepressants	145 (41.9%)	N/A		
Benzodiazepine and anxiolytics	105 (30.3%)	N/A		
Hypnotics/sedatives	37 (10.7%)	N/A		
Others	48 (13.9%)	N/A		
**BDI-II**				
Total depression score	30.83 (10.28)	3.85 (3.31)	1104.99***	<0.001
Cognitive depression	22.74 (8.65)	2.15 (2.29)	938.95***	<0.001
Somatic depression	8.09 (3.30)	1.71 (1.61)	530.45***	<0.001
**Breathing rates**	15.55 (3.72)	14.18 (3.71)	10.53**	0.001
**Heart rate (bpm)**	73.76 (10.64)	71.05 (8.59)	6.30*	0.013
**HRV indices**				
SDNN (ms)	32.95 (15.81)	41.47 (14.60)	27.14***	<0.001
LF (ms^2^/Hz)	109.39 (250.66)	196.45 (219.82)	11.83***	<0.001
lnLF	3.86 (1.28)	4.74 (1.09)	47.41***	<0.001
HF (ms^2^/Hz)	118.39 (153.22)	160.57 (141.14)	7.10**	0.008
lnHF	4.01 (1.38)	4.61 (1.11)	19.81***	<0.001
LF/HF ratio	1.87 (4.51)	2.31 (4.08)	0.91	0.342
**CVC indices**				
RSA (ms^2^/Hz)	88.66 (123.01)	114.92 (110.18)	4.32*	0.038
lnRSA	3.68 (1.40)	4.29 (1.05)	21.09***	<0.001

*p < 0.05, **p < 0.01, ***p < 0.001.

*BDI-II, Beck Depression Inventory II; CVC, cardiac vagal control; HC, healthy controls; HF, high frequency; LF, low frequency; lnLF, natural logarithm of low frequency; lnHF, natural logarithm of high frequency; lnRSA, natural logarithm of respiration sinus arrhythmia; MDD, major depressive disorder; RSA, respiration sinus arrhythmia; SDNN, standard deviation of all normal to normal intervals*.

There were higher total depression scores, cognitive depression, and somatic depression in the MDD group than in the HC group (*F* = 1104.99, *p* < 0.001; *F* = 938.95, *p* < 0.001; and *F* = 530.45, *p* < 0.001, respectively), as well as higher breathe rate and heart rate in the MDD group than in the HC group (*F* = 10.53, *p* = 0.001 and *F* = 6.30, *p* = 0.013, respectively). However, there was a lower SDNN, LF, lnLF, HF, and lnHF of cardiac autonomic regulation (*F* = 27.14, *p* < 0.001; *F* = 11.83, *p* < 0.001; *F* = 47.41, *p* < 0.001; *F* = 7.10, *p* < 0.01; and *F* = 19.81, *p* < 0.001, respectively) and lower RSA and lnRSA of CVC (*F* = 4.32, *p* = 0.038; *F* = 21.09, *p* < 0.001, respectively) in the MDD group than in the HC group. There was no significant difference in the LF/HF ratio between the two groups (*F* = 0.91, *p* = 0.342) (Table 1).

### The Differences Between Experimental Stages in HRV and CVC Indices for the MDD and HC Groups

In the MDD group, there was a significant time main effect in lnLF (*F* =47.78, *p* < 0.001, $\eta_{\text{p}}^{2}$ = 0.26). The Bonferroni *post-hoc* exam found a higher lnLF during the baseline than that during depressive autobiographical memory (B > D), as well as higher lnLF during depressive recovery than during depressive autobiographical memory (DR > D), and higher lnLF during happiness recovery than during happiness autobiographical memory (HR > H).

**Figure 2:**
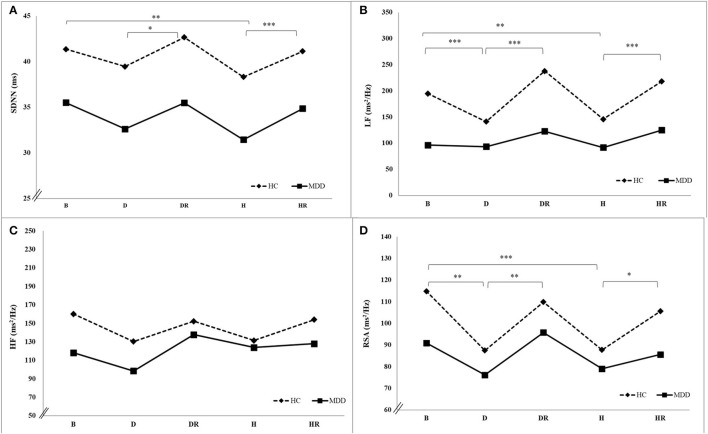
HRV **(A–C)** and CVC **(D)** indices under depressive and happiness autobiographical memories for the MDD and HC groups. B, baseline; D, depressive autobiographical memory; DR, depressive recovery; H, happiness autobiographical memory; HR, happiness recovery.

In the HC group, there were significant time main effect in SDNN, LF, lnLF, LF/HF ratio, RSA, and lnRSA (*F* = 3.32, *p* < 0.05, $\eta_{\text{p}}^{2}$ = 0.02; *F* = 8.29, *p* < 0.001, $\eta_{\text{p}}^{2}$ = 0.05; *F* = 142.84, *p* < 0.001, $\eta_{\text{p}}^{2}$ = 0.45; *F* = 13.94, *p* < 0.001, $\eta_{\text{p}}^{2}$ = 0.07; *F* = 5.45, *p* < 0.001, $\eta_{\text{p}}^{2}$ = 0.03; and *F* = 4.30, and *p* < 0.01, $\eta_{\text{p}}^{2}$ = 0.03, respectively). The Bonferroni *post-hoc* examination found a higher SDNN during the baseline than during happiness autobiographical memory (B > H), higher SDNN during depressive recovery than during autobiographical memory (DR > D), and higher SDNN during happiness recovery than during happiness autobiographical memory (HR > H). There were higher LF, lnLF, RSA power, and lnRSA during baseline than during depressive autobiographical memory and happiness autobiographical memory (B > D and B > H), as well as higher LF, lnLF, RSA, and lnRSA during depressive recovery than during depressive autobiographical memory (DR > D), and higher LF, lnLF, RSA, and lnRSA during happiness recovery than during happiness autobiographical memory (HR > H) (Table 2, Figure 2).

**Table 2:** One-way repeated measure ANOVA at different experimental stages between the MDD and HC groups in HRV and CVC indices.

**Variables**	**Time**								
		**Baseline (B)**	**Depressive autobiographical memory (D)**	**Depressive recovery (DR)**	**Happiness autobiographical memory (H)**	**Happiness recovery (HR)**	***F***	***ηp^**2**^***	**Bonferroni Post-hoc comparison**
**HRV indices**								
SDNN (ms)	MDD	35.52 (15.52)	32.62 (16.66)	35.48 (18.72)	31.45 (16.91)	34.86 (18.54)	1.04	0.01	
	HC	41.37 (14.59)	39.46 (15.95)	42.67 (16.78)	38.32 (13.84)	43.13 (15.67)	3.32*	0.02	**B>H, DR>D, HR>H** DR>H, HR>D
LF (ms^2^/Hz)	MDD	96.30 (133.51)	93.26 (131.52)	122.62 (199.72)	91.55 (143.65)	124.88 (182.45)	1.21	0.01	
	HC	194.88 (219.44)	141.20 (149.37)	237.91 (289.65)	145.94 (178.72)	218.04 (252.48)	8.29***	0.05	**B>H, B>D, HR>H, DR>D**, DR>H, HR>D
lnLF	MDD	3.85 (1.27)	1.30 (0.37)	4.05 (1.25)	3.82 (1.21)	4.07 (1.27)	47.78***	0.26	**B>D, H>D, HR>H, DR>D, HR>B**, DR>H, HR>D,
	HC	4.73 (1.09)	1.47 (0.27)	4.89 (1.14)	4.47 (1.03)	4.84 (1.08)	142.84***	0.45	**B>H, B>D, H>D, HR>H, DR>D**, DR>H, HR>D, DR>B,
HF (ms^2^/Hz)	MDD	118.17 (162.12)	98.57 (128.52)	137.71 (257.18)	123.90 (208.77)	128.02 (210.45)	0.86	0.01	
	HC	160.17 (141.44)	130.30 (120.46)	152.34 (144.86)	131.51 (120.85)	154.00 (151.96)	0.70	<0.01	
lnHF	MDD	3.97 (1.38)	3.79 (1.43)	4.03 (1.38)	3.93 (1.40)	4.08 (1.28)	0.39	<0.01	
	HC	4.60 (1.11)	4.39 (1.11)	4.57 (1.09)	4.41 (1.08)	4.59 (1.03)	0.43	<0.01	
LF/HF ratio	MDD	1.88 (4.86)	1.95 (2.97)	1.58 (2.29)	1.40 (2.00)	1.64 (2.80)	0.18	<0.01	
	HC	2.31 (4.09)	1.74 (2.08)	2.45 (3.42)	1.76 (2.67)	2.35 (3.44)	13.94***	0.07	DR>D
**CVC index**								
RSA (ms^2^/Hz)	MDD	90.91 (131.41)	76.15 (113.81)	95.75 (151.58)	79.01 (120.86)	85.62 (140.67)	0.26	<0.01	
	HC	114.86 (110.50)	87.47 (77.92)	109.90 (98.41)	87.75 (86.51)	105.63 (94.36)	5.45***	0.03	**B>D, B>H, DR>D, HR>H**, HR>D, DR>H
lnRSA	MDD	3.65 (1.42)	3.51 (1.38)	3.70 (1.39)	3.53 (1.39)	3.65 (1.33)	0.37	<0.01	
	HC	4.28 (1.05)	4.03 (1.07)	4.27 (1.06)	4.01 (1.06)	4.25 (1.00)	4.30**	0.03	**B>H, B>D, HR>H, DR>D**, DR>H, HR>D
Breathing	MDD	15.57 (3.73)	15.33 (3.63)	15.17 (3.27)	15.73 (3.16)	15.49 (3.14)	**2.26**	0.02	
rate (bpm)	HC	14.18 (3.71)	15.16 (5.72)	14.35 (3.74)	15.54 (3.73)	14.50 (3.74)	8.86***	**0.05**	**B < H, H>HR** DR < H

*p <0.05, **p <0.01, ***p <0.001.

*B, baseline; CVC, cardiac vagal control; D, depressive autobiographical memory; DR, depressive recovery; H, happiness autobiographical memory; HC, healthy controls; HF, high frequency; HR, happiness recovery; LF, low frequency; lnLF, natural logarithm of low frequency; lnHF, natural logarithm of high frequency; lnRSA, natural logarithm of respiration sinus arrhythmia; MDD, major depressive disorder; RSA, respiration sinus arrhythmia; SDNN, standard deviation of all normal to normal intervals.*

*The bold values used to indicate the significant differences between five experimental stages*.

### The HRV/RSA Reactivity of Depressive/Happiness Autobiographical Memory Tasks and Baseline Between the MDD and HC Groups

HRV/RSA reactivity of happiness autobiographical memory (H) and baseline (B) significantly decreased in ΔHF in the HC group compared to the MDD group (*F* = 7.81, *p* < 0.01); however, there was not a significant difference in HRV/RSA reactivity of depressive autobiographical memory (D) and baseline between the two groups (Table 3).

**Table 3:** The HRV reactivity and HRV recovery between the MDD and HC groups.

**Variables**	**MDD group**	**HC group**	***F***	***p***	**Bonferroni Post–hoc comparison**
**HRV reactivity** **=** **depressive autobiographical memory (D) – baseline (B)**					
ΔSDNN	0.38 (11.35)	−1.86 (13.36)	2.81	0.094	
ΔLF	−11.2 (216.73)	−52.61 (173.67)	3.86	0.050	
ΔHF	−19.98 (86.76)	−29.96 (89.5)	1.11	0.294	
ΔRSA	−14.30 (56.23)	−27.21 (92.68)	2.40	0.122	
**HRV reactivity** **=** **happiness autobiographical memory (H) – baseline (B)**					
ΔSDNN	−1.87 (10.07)	−3.02 (11.01)	1.02	0.313	
ΔLF	−18.97 (190.3)	−47.83 (191.89)	1.96	0.162	
ΔHF	0.48 (101.71)	−29.04 (94.74)	7.81**	0.005	HC > MDD
ΔRSA	−12.66 (73.24)	−26.85 (73.74)	3.18	0.076	
**HRV recovery** **=** **depressive recovery (DR) – depression autobiographical memory (D)**					
ΔSDNN	2.27 (9.57)	3.18 (13.56)	0.52	0.472	
ΔLF	22.5 (133.24)	96.66 (234.48)	12.82***	<0.001	HC > MDD
ΔHF	34.59 (159.16)	22.3 (97.92)	0.76	0.385	
ΔRSA	16.30 (89.18)	22.40 (74.28)	0.47	0.493	
**HRV recovery** **=** **happiness recovery (HR) – happiness autobiographical memory (H)**					
ΔSDNN	3.91 (8.24)	4.84 (11.24)	0.75	0.386	
ΔLF	35.47 (118.29)	73.92 (214.36)	4.18*	0.042	HC > MDD
ΔHF	4.24 (62.01)	23.6 (83.74)	5.91*	0.016	HC > MDD
ΔRSA	6.53 (47.89)	18.30 (71.01)	3.20	0.074	
**HRV recovery** **=** **depressive recovery (DR) – baseline (B)**					
ΔSDNN	2.65 (11.6)	1.32 (10.99)	1.19	0.275	
ΔLF	11.3 (246.86)	44.05 (256.34)	1.46	0.228	
ΔHF	14.61 (189.19)	−7.65 (82.71)	2.05	0.153	
ΔRSA	2.00 (89.52)	−4.81 (71.60)	0.60	0.438	
**HRV recovery** **=** **happiness recovery (HR) – baseline (B)**					
ΔSDNN	2.05 (10.52)	1.82 (9.7)	0.04	0.837	
ΔLF	16.5 (171.52)	26.09 (234.81)	0.19	0.667	
ΔHF	4.72 (121.56)	−5.44 (81.75)	0.84	0.360	
ΔRSA	−6.13 (92.19)	−8.55 (73.70)	0.07	0.789	

*p <0.05, **p <0.01, ***p <0.001.

*CVC, cardiac vagal control; RSA, respiration sinus arrhythmia; SDNN, standard deviation of all normal to normal intervals; LF, low frequency; HC, healthy controls; HF, high frequency; MDD, major depressive disorder; Δ reactivity and recovery*.

### The HRV/RSA Recovery of Depressive/Happiness Autobiographical Memory Tasks and Depressive/Happiness Recovery Between the MDD and HC Groups

**Figure 3:**
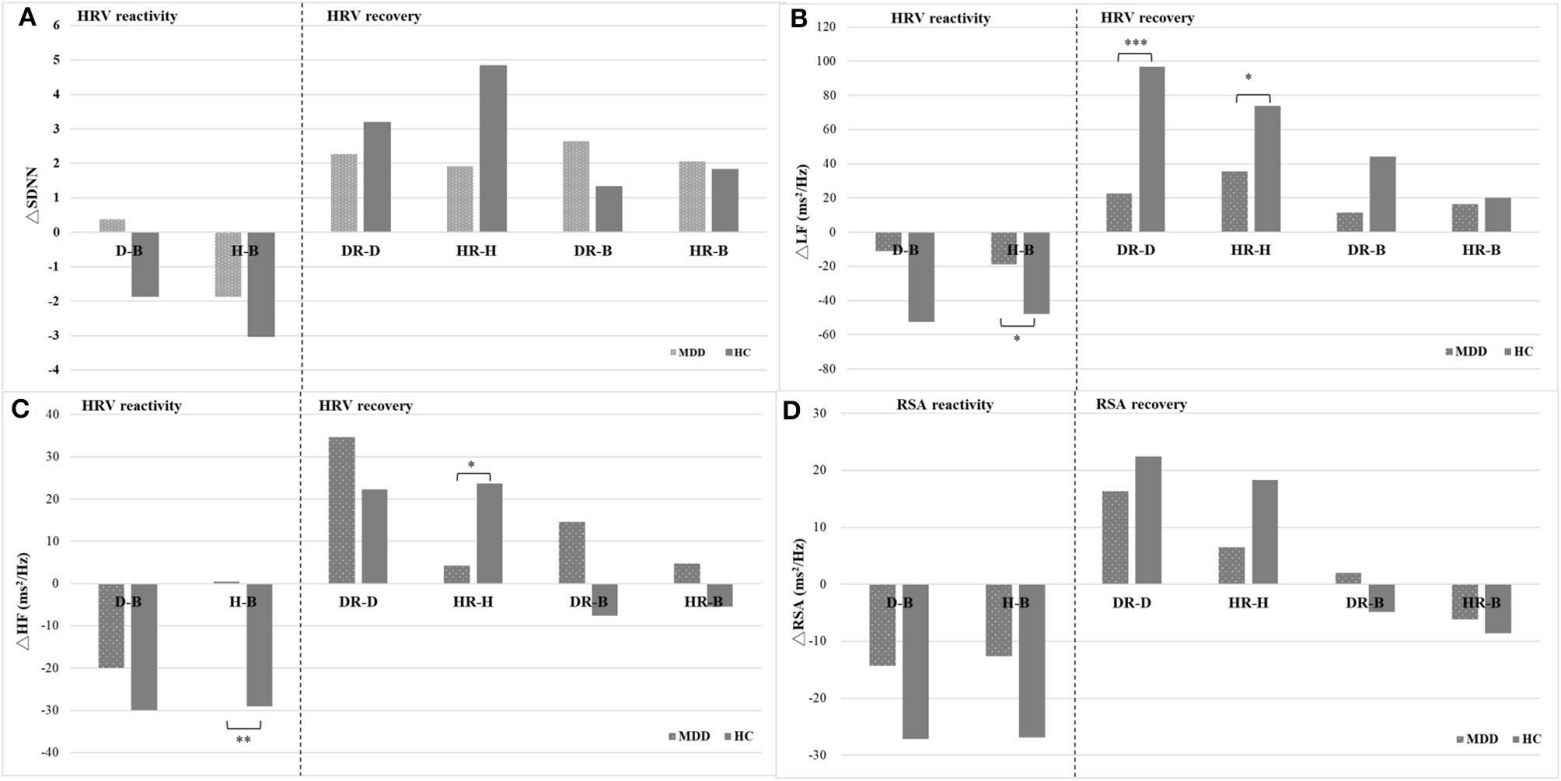
The reactivity and recovery of cardiac autonomic **(A–C)** and CVC **(D)** for the MDD and HC groups. B, baseline; D, depressive autobiographical memory; DR, depressive recovery; H, happiness autobiographical memory; HR, happiness recovery.

There was a significant increase in HRV/RSA recovery of depressive autobiographical memory and depressive recovery in ΔLF in the HC group compared to the MDD group (*F* = 12.82, *p* < 0.001), as well as a significant increase in HRV recovery of happiness autobiographical memory and happiness recovery in ΔLF and ΔHF in the HC group compared to the MDD group (*F* = 4.18, *p* < 0.05 and *F* = 5.91, *p* < 0.05, respectively). There were no significant differences in depressive recovery (DR) and baseline (B) as well as happiness recovery (HR) and baseline (B) between the MDD and HC groups (Table 3, Figure 3).

### Correlations Between Depressive Symptoms and Indices of Cardiac Autonomic Activation and CVC in All Participants

There were positive correlations between total depression score, cognitive depression, and heart rate (*r* = 0.12, *p* < 0.05 and *r* = 0.12, *p* < 0.05), and negative correlations between total depression score, cognitive depression, somatic depression, and the indices of HRV and CVC, including SDNN, LF, lnLF, lnHF, and lnRSA power (*r* = −0.14 to −0.32). However, there was no significant correlation between the total depression score, cognitive depression, somatic depression, HF, LF/HF ratio, and RSA power (Table 4).

**Table 4:** The correlations between depression and baseline HRV and CVC indices.

**Variables**		**HRV indices**						**CVC indices**	
	**heart rate**	**SDNN**	**LF**	**lnLF**	**HF**	**lnHF**	**LF/HF ratio**	**RSA**	**lnRSA**
Total depression score	0.12*	−0.21***	−0.19**	−0.32***	−0.07	−0.16**	−0.06	−0.03	−0.15**
Cognitive depression	0.12*	−0.21***	−0.20**	−0.31***	−0.06	−0.15**	−0.07	−0.03	−0.14*
Somatic depression	0.10	−0.19**	−0.14*	−0.29***	−0.08	−0.15**	−0.03	−0.02	−0.14*

*p <0.05, **p <0.01, ***p <0.001.

*CVC, cardiac vagal control; HF, high frequency; LF, low frequency; lnLF, natural logarithm of low frequency; lnHF, natural logarithm of high frequency; lnRSA, natural logarithm of respiration sinus arrhythmia; RSA, respiration sinus arrhythmia; SDNN, standard deviation of all normal to normal intervals*.

## Discussion

The most significant findings included lower cardiac autonomic and CVC during the resting baseline in the MDD group compared to the HC group. Participants in the MDD group showed reduced reactivity and recovery of cardiac autonomic and CVC during and after depressive and happiness autobiographical memory tasks compared to the HC group. Depressive symptoms were positively correlated with the heart rate and negatively correlated with the indices of cardiac autonomic and CVC.

The reduced cardiac autonomic and CVC during the resting baseline in patients with MDD were consistent with previous studies that lower HRV was found in patients with first-episode MDD (2), unmedicated depression (24), late-life depression (16, 18, 35), and childhood and adolescent depression (36). These results revealed reduced vagal and parasympathetic activations and cardiac autonomic and showed higher HR and LF/HF ratio, lower SDNN, LF, HF, and lnRSA during the resting baseline. The psychophysiological mechanism indicated vagal and parasympathetic withdrawal in the MDD group (11, 28) and made it harder for participants with MDD to restore or replenish psychological or physical energy (4).

Regarding the reactivity of cardiac autonomic and CVC, participants in the MDD group did not demonstrate the flexibility to regulate their cardiac autonomic and CVC. Our results showed that participants in both groups had decreased HF of HRV during depressive autobiographical memory, with no group difference. However, there was a significant decrease in vagal and PNS activity (HF) during happiness autobiographical memory in the HC group compared to that in the MDD group. Therefore, this result supported the vagal tank theory, participants in the HC group had better self-regulation with larger PNS and CVC decreases during happiness autobiographical memory (17). Moreover, patients with MDD have a deficit in positive autobiographical memory (28, 32), and this makes it hard to regulate their PNS and CVC to cope with happiness autobiographical memory (27). Reduced flexibility in the PNS and CVC is related not only to the severity of depression but also to hypoactivity in the amygdala, which is an important brain area for emotional regulation (32).

Regarding the recovery of cardiac autonomic and CVC, the participants in the HC group recovered more magnitude or rapidly in cardiac autonomic (LF) after depressive and happiness autobiographical memories in the HC group compared to the MDD group, which presented healthy participants with the flexibility to regulate their cardiac autonomic. The physiological mechanism of LF is influenced by both SNS and PNS, and LF is also modulated by the vagal pathway through the baroreflex receptor to regulate blood pressure. Activated CVC can balance the SNS and PNS and regulate cardiac rhythm, heart rate, and cardiac output. Moreover, activated baroreflex receptors can regulate blood pressure and restore the heart rate and breathing patterns to homeostasis (17, 31). In addition, participants in the HC group recovered more in their CVC (HF of HRV) after the happiness autobiographical memory task compared to the MDD group. Interestingly, participants in the two groups did not show a difference in CVC after the depressive autobiographical memory, indicating that the CVC was influenced by depressive events and needed to take longer or slower to recover to the initial baseline. According to vagal rebound and vagal tank theory, recovering more or faster indicates better self-regulation and emotional adaptation (26, 31). The results were consistent with the results of Rottenberg et al. (26), who found increased CVC after the sad films in the HC group, but not in the MDD group. Rottenberg et al. (26) indicated vagal rebound, which is a homeostatic function or buffering effect of RSA for increasing PNS tone both during and after the sad film. Low RSA or HRV in the MDD group may increase the risk of cardiac morbidity or mortality (38). RSA increases under stress situations (speech task and mirror tracking task) or sad film in the HC group and recovers rapidly in the CVC after stress. However, this effect was not observed in the MDD group. The possible reason was that patients with MDD experienced depressive emotions repeatedly, decreased flexibility or fluctuation in their CVC, or vagal withdrawal (22). While CVC is related to individuals' ability and flexibility under social interaction or stress situations, increasing RSA fluctuation under stress situations is a buffering effect that increases individuals' behavioral flexibility and adaptation (26). Low RSA levels and blunted RSA reactivity are related to impaired emotion regulation capacity in participants with MDD (27).

This study was supported by a positive correlation between depressive severity and heart rate and SNS hyperactivity (34), and negative correlations between depressive severity and cardiac autonomic and CVC (12), which indicated PNS or vagal withdrawal. This study extended the depressive severity supported by cognitive depression, and somatic depression was also related to poor cardiac autonomic function and CVC (33).

This study has two limitations. First, participants were not asked to suspend prescriptions before ECG measurements, and a previous study reported that antidepressants (such as tricyclic) may decrease HRV values (8, 35). Second, this study had a cross-sectional research design, depressive symptoms were correlated with cardiac autonomic and CVC, and longitudinal studies and follow-up will be needed in future studies.

## Conclusion

This study confirmed cardiac autonomic dysregulation and vagal withdrawal in patients with MDD. There were reduced large cardiac autonomic and CVC and active vagal rebounds to recover to the initial baseline during and after the happiness autobiographical memory task in the HC group, but not in the MDD group. HRV biofeedback with slow-paced breathing has been confirmed to restore cardiac autonomic regulation and CVC during the resting baseline in participants with MDD (36–38). Future studies will explore the efficacy of HRV biofeedback in HRV/CVC reactivity and recovery for participants with MDD.

## Data Availability Statement

The raw data supporting the conclusions of this article will be made available by the authors, without undue reservation.

## Ethics Statement

The studies involving human participants were reviewed and approved by Ethics Committee of Kaohsiung Medical University Hospital, Taiwan. Ethics Committee of Kaohsiung Chang Gung Memorial Hospital, Taiwan. The patients/participants provided their written informed consent to participate in this study.

## Author Contributions

Y-CW conducted the original study and performed the HRV and statistical analyses. P-YL, C-LKK, M-FH, Y-CY, K-TW, C-FY, and C-HK recruited and referred the study participants. I-ML was responsible for the planning and conducting of the original study and supervised the ECG recordings and HRV analysis. W-SS checked draft of the manuscript. I-ML and S-YF drafted and revised the manuscript accordingly. All authors participated substantially in writing the manuscript. All authors contributed to the article and approved the submitted version.

## Funding

I-ML received a research grant from the Ministry of Science and Technology, Taiwan, and the grant numbers are 101-2410-H-037-007, 105-2410-H-037-002, 106-2410-H-037-003, and 110-2410-H-037-003. The funding sources had no role in the design and conduct of the study, preparation, review, or approval of the manuscript.

## Conflict of Interest

The authors declare that the research was conducted in the absence of any commercial or financial relationships that could be construed as a potential conflict of interest.

## Publisher's Note

All claims expressed in this article are solely those of the authors and do not necessarily represent those of their affiliated organizations, or those of the publisher, the editors and the reviewers. Any product that may be evaluated in this article, or claim that may be made by its manufacturer, is not guaranteed or endorsed by the publisher.
